# 1173. Changes in Invasive Pneumococcal Disease Incidence Following Introduction of PCV10 and PCV13 Among Children < 5 Years: The PSERENADE Project

**DOI:** 10.1093/ofid/ofab466.1366

**Published:** 2021-12-04

**Authors:** Julia C Bennett, Maria Deloria Knoll

**Affiliations:** Johns Hopkins Bloomberg School of Public Health, Baltimore, Maryland

## Abstract

**Background:**

Higher valency pneumococcal conjugate vaccines (PCV10 and PCV13) replaced PCV7, and an updated global analysis of PCV impact on invasive pneumococcal disease (IPD) incidence is needed. We aimed to estimate the change in vaccine-type (VT), non-VT type and all-serotype (ST) IPD incidence following introduction of PCV10/13 among children < 5 years of age.

**Methods:**

IPD ST-specific incidence or cases and population denominators were obtained directly from surveillance sites. IPD incidence rate ratios (IRRs) for each site were estimated comparing the pre-any PCV incidence to each post-PCV10/13 year using Bayesian multi-level, mixed effects Poisson regressions. All-site weighted average IRRs were estimated using linear mixed-effects regressions. Results were stratified by product (PCV10 vs. PCV13) and years of prior PCV7 use (none, some [1-3 years or 4-5 years if < 70% PCV uptake], or many [≥ 4 years with ≥ 70% uptake]).

**Results:**

Analyses included 45 surveillance sites from 31 countries, primarily high-income (80%). Thirty surveillance sites had pre- and post-PCV data (PCV10: no prior PCV7=5 sites, some=2, many=2; PCV13: no prior PCV7=3, some=5, many=13). Five years after PCV10/13 introduction, the all-site IRRs in children < 5 years were generally similar across products and prior PCV7 use strata for all-serotype IPD (range 0.23-0.41), PCV7 STs (0.01-0.13), PCV10non7 STs (1, 5, and 7F; 0.05-0.20), and ST6A (0.01-0.18). IRRs for ST19A were lower for PCV13 sites (range by PCV7 use: 0.09-0.31) than for PCV10 sites (1.1-1.4). ST3 IRRs were dynamic, differing by product at year 5 (range for PCV13 sites=0.86-1.02; PCV10 sites=1.55-1.78), but converging by year 7. NonPCV13 STs increased across all strata (range 1.9-2.6), except one strata with a single African site that declined.

Figure 1. All-Site Weighted Average Incidence Rate Ratios, Children <5>

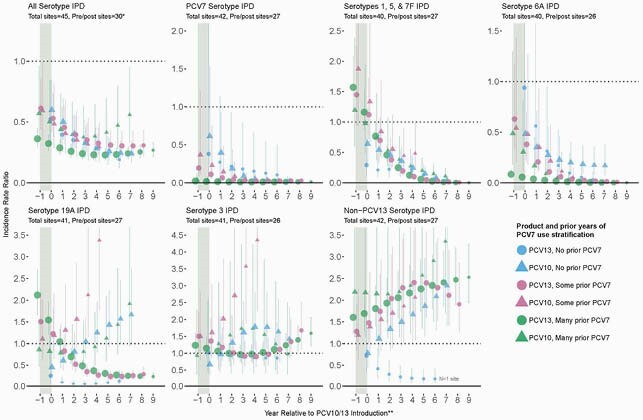

* Total sites indicates number of sites with incidence rate data included and pre/post sites indicates number of sites with both pre- and post-PCV data to estimate IRRs for each outcome. ** Year 0 indicates the year of PCV10/13 introduction and year -1 indicates the last year of PCV7 use prior to PCV10/13 introduction.

**Conclusion:**

All-serotype IPD in children < 5 years declined following both PCV10 and PCV13 use, driven by substantial declines in VT serotypes and offset by increases in nonPCV13 STs. ST19A decreased among PCV13-sites, mitigating replacement disease occurring after PCV7 use, but increased, on average, among PCV10-sites. Changes in ST3 were heterogeneous, increasing in some sites and no change from baseline in others. Data from low-income and high-burden settings were limited.

**Disclosures:**

**Julia C. Bennett, MSPH**, **Pfizer** (Research Grant or Support) **Maria Deloria Knoll, PhD**, **Merck** (Research Grant or Support)**Pfizer** (Research Grant or Support)

